# Distal Radius Reconstruction Using Proximal Non-vascularized Fibula Graft in a Patient During the Syrian Conflict: A Case Study of Treatment Outcomes

**DOI:** 10.7759/cureus.55821

**Published:** 2024-03-08

**Authors:** Mohamad Khatib, Ibrahim W Hasani

**Affiliations:** 1 Surgery, Idlib University Hospital, Idlib, SYR; 2 Biochemistry, Idlib University Hospital, Idlib, SYR

**Keywords:** case report, non-vascularized fibula graft, syrian conflict, radius, benign bone tumor, giant cell tumor

## Abstract

In conflict zones like Syria, accessing specialized medical care presents significant challenges. Here, we present the case of a 22-year-old female with a giant cell tumor in her distal forearm, exacerbated by limited access to healthcare due to the Syrian conflict. Despite these obstacles, we successfully performed en bloc resection and reconstructed the defect with a proximal non-vascularized fibular graft, restoring arm function. This case underscores the critical importance of adapting to adverse circumstances to deliver essential medical interventions in conflict-affected regions.

## Introduction

Giant cell tumor (GCT) of bone is a primary bone tumor that is benign but aggressive. It is the most common type of such tumors and accounts for approximately 5% of primary bone tumors in adults aged 20-50 years [1]. It tends to affect females more than males [2]. In less than 5% of cases, GCT can metastasize, most often to the lungs, although the course is generally indolent, and fatality is rare [3].

The radiographic appearance of GCT is typically an eccentrically located lesion in the metaphyseal and epiphyseal regions of long bones. The hallmark appearance is an expansile lesion that is centrally radiolucent, with the formation of a thin neocortex at the border. The Campanacci grade is used to classify GCTs, with grade 1 tumors confined within the cortex, grade 2 expanding the cortex, and grade 3 perforating the cortex with resultant soft tissue extension. GCTs are most commonly found in the knee region (distal femur and proximal tibia), with only 10% of cases occurring in the distal radius [4,5].

Surgery is the mainstay of treatment for GCT, with options including en bloc resection and reconstruction or intralesional curettage with a high-speed burr, cryotherapy or phenolization, and cementation or bone grafting [6]. The surgical treatment of bone tumors has been a topic of controversy. It is essential to remove tumor tissue adequately, but preserving limb function is equally important. In cases of well-contained GCTs, extended curettage yields satisfactory outcomes. However, for tumors that have cortical breaches or large soft tissue extensions, en bloc resection is necessary. This involves the removal of the tumor and reconstruction of the defect using an endoprosthesis to restore joint and limb function [7].

The Syrian conflict has had a significant impact on the provision of medical care, including the surgical treatment of patients with grade III GCTs of the distal radius. In this context, the management of a distal forearm lump caused by a large GCT poses unique challenges due to the limited resources and decreased availability of medical devices and expert personnel. Despite these difficulties, it is crucial to provide appropriate and timely surgical intervention to prevent the progression of the disease and preserve the patient's wrist function. As a result, the surgical team may need to resort to alternative methods and techniques to perform the surgery, such as using locally available materials and adapting standard surgical protocols to the available resources.

We present a unique case of a distal forearm lump caused by a large GCT of the distal radius. Definitive management consisted of wide en bloc resection reconstruction with the non-vascularized proximal fibula, along with the shortening of the distal ulna, with local control and good clinical outcomes.

This article was previously posted in the Research Square preprint server on March 4, 2023 (https://doi.org/10.21203/rs.3.rs-2675256/v1).

## Case presentation

**Figure 1:**
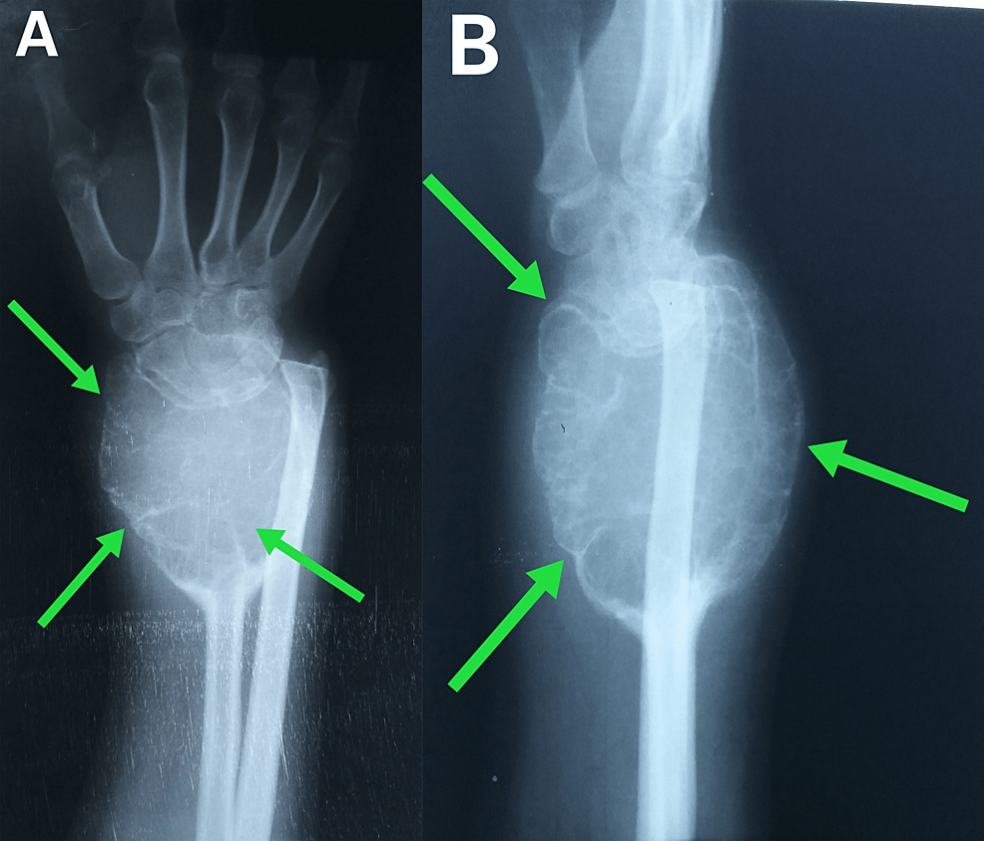
Pre-operative anteroposterior (A) and lateral (B) X-rays of the wrist. The arrows indicate a huge lobulated lesion that invades the distal third of the radius and surrounds the dorsal and palmar aspects of the proximal row of the wrist bones. The lesion causes medial dislocation and elongation of the ulna.

A 22-year-old female presented with a progressively increasing painful and tender lump in the distal forearm, along with restricted wrist movements and inability to use her right hand. Physical examination showed a large lump in the distal radius and severely restricted pronation and supination, as well as limited dorsal extension and palmar flexion of the wrist, and fully restricted radial and ulnar deviations. Anterioposterior and lateral X-ray revealed a large multilocular radiolucency with thin walls, resulting in medial ulnar head dislocation. A delay in diagnosis caused a change in the radioulnar index and an increase in the length of the dislocated ulna compared to the radius (Figure 1). Laboratory tests were normal, and metastasis was ruled out using chest X-ray and computerized tomography (CT) due to its 3% occurrence rate.

The patient underwent a CT scan, which revealed a large lobulated expansile mass with central necrotic hemorrhagic areas measuring 10 cm in proximodistal dimension and about 7 cm in lateromedial dimension. The mass had a very thin wall. The biopsy revealed type I cell mononuclear stromal cell resembling interstitial fibroblasts, type II cell from monocyte/macrophage family recruited from peripheral blood precursors of giant cells, and numerous giant cells as type III cell, with secondary ABC degeneration in the sections. Based on these findings, the operation planned to perform entire lesion resection and reconstruction with the non-vascularized proximal fibula, along with the shortening of the distal ulna.

Harvesting of non-vascularized proximal fibular graft

**Figure 2:**
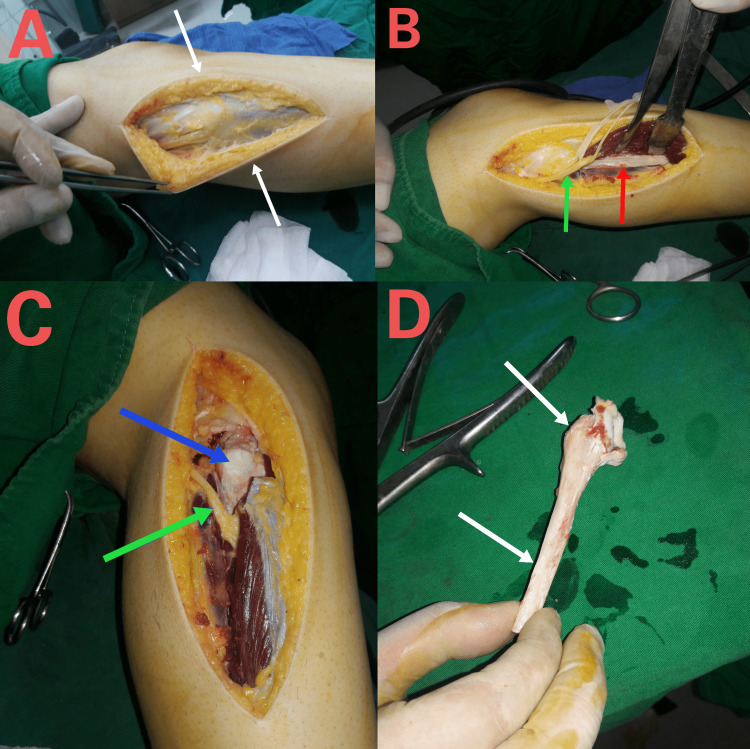
Harvest of the graft. (A) Lateral approach of the proximal fibula; (B) Isolation of deep peroneal nerve (green arrow) and sub-periosteally exposure of proximal fibula (red arrow); (C) Detachment of all tendons and ligaments of the fibular head(blue arrow); (D) Proximal tibiofibular joint disarticulation and osteomize fibula 10 cm distal to the apex of the fibular head to harvest the graft (yellow arrow).

Harvesting the non-vascularized proximal fibular graft involved several steps. Firstly, a 15 cm curved and longitudinal skin incision was made, approximately parallel to the fibula. This incision was centered on the fibular head, starting 7 cm above it and about 1 cm behind the fibula. Extending towards the distal fibula, it concluded in the proximal one-third of the leg. During this process, special attention was given to exposing and protecting the common peroneal nerve, superficial peroneal nerve, and deep peroneal nerve. Subsequently, the detachment of the biceps femoris tendon and fibular collateral ligament from the fibular head was performed. Osteomization of the fibula occurred 10 cm distal to the fibular head tip, resulting in the harvesting of the proximal fibula (Figure 2). The length of the fibular graft was determined by the bone defect resulting from the distal radius resection. The final step involved the reconstruction of the remaining soft tissue, including the biceps femoris tendon, fibular collateral ligament, and proximal tibiofibular joint capsule, to preserve the lateral stability of the knee.

En bloc resection of distal radius

Enbloc resection of the distal radius involved a dorsal skin incision. The tumor was dissected extra-periosteally, and special care was taken to isolate and protect the extensor tendons, neurovascular bundles, and flexor tendons. Osteotomization of the radius occurred 1cm proximal to the tumor. The final step involved the disarticulation of the radio-carpal joint by circumferentially detaching the entire wrist capsule and ligaments.

Distal ulna shortening

**Figure 3:**
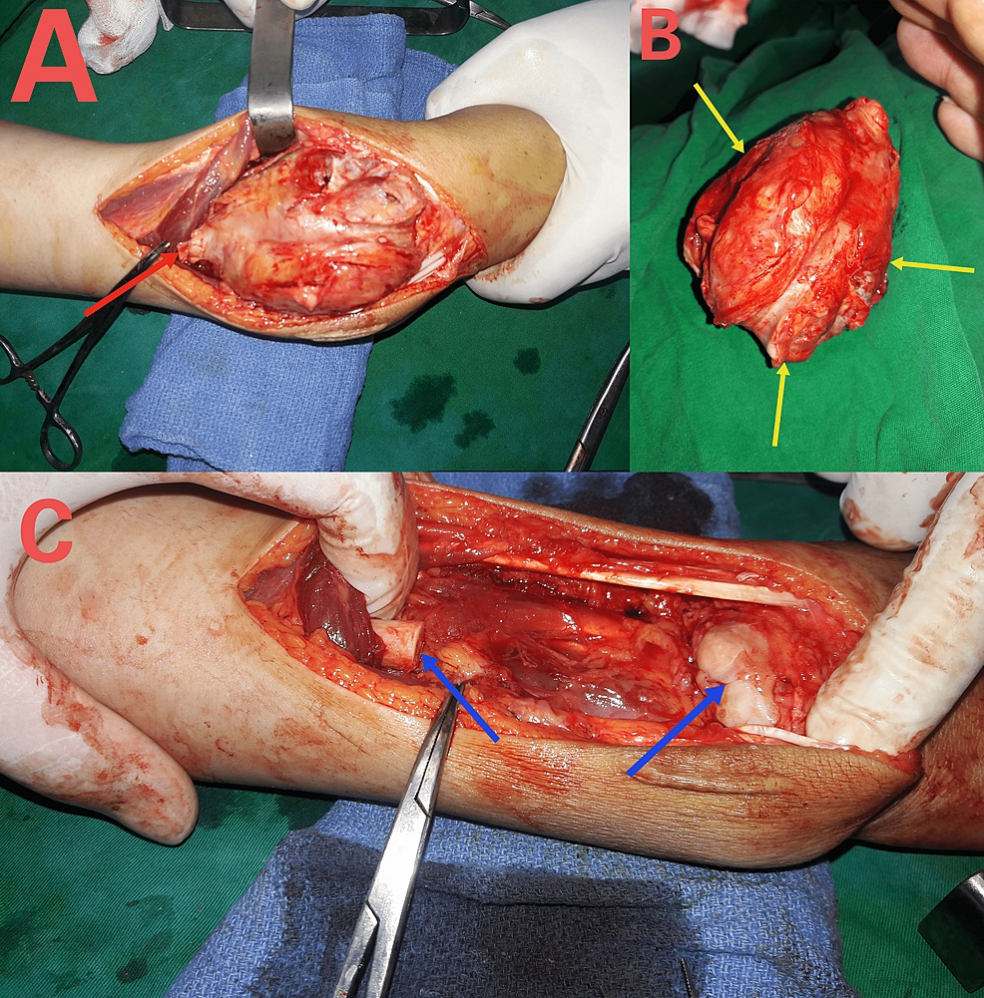
Tumor resection. (A) Dorsal approach of the distal radius curved distally to the ulnar head, osteomization of radius proximal to the tumor (red arrow) and isolation of tendons, median nerve, radial artery away from the tumor and wrist joint disarticulation; (B) Resection of tumor (10×8×7cm) (Yellow arrows); (C) Bone defection after resection (blue arrows).

Because of severe positive radio-ulnar variance, we shortened the distal ulna 1 cm to restore the distal radio-ulnar joint and stabilized osteotomy with DCP (Figure 3).

Reconstruction of the distal radius

**Figure 4:**
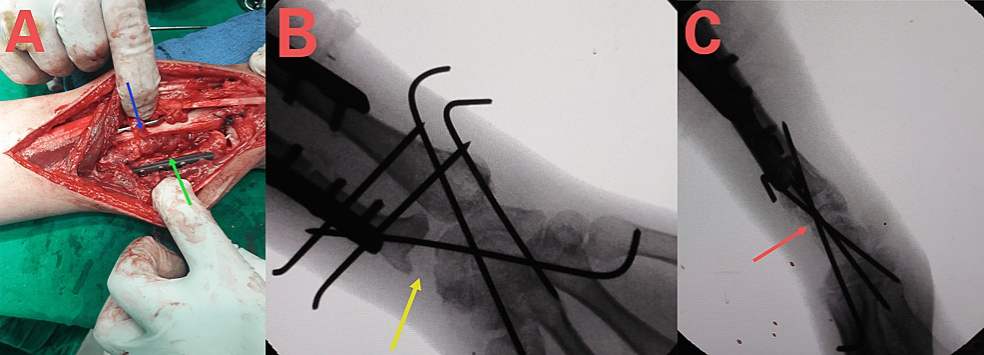
Final recontraction. (A) Stabilization of fibular graft in its place by DCP plate (blue arrow) and suturing of the wrist capsule to bony tunnels in the fibular head and with residual soft tissues attached to the fibular head, ulnar shortening (green arrow) to restore radio-ulnar variance, securing the bones to each other's by K-wires; (B) Anteroposterior view during operation (yellow arrow); (C) Lateral view during operation (red arrow). K-wire: Kirschner wire; DCP: dynamic compression plate

In reconstructing the distal radius, fine tunnels were prepared at the dorsal and anterior edges of the fibular head for securing it to the dorsal and palmar aspects of the wrist capsule. Proximal stabilization of the fibular graft was achieved using a dynamic compression plate (DCP), while distal fixation to the carpal bones employed two Kirschner wire (K-wire) positioned at a functional angle (20° of wrist extension). The palmar and dorsal capsules were sutured to the edges of the fibular head. Subsequently, the shortened ulna and new radius were stabilized using transverse two K-wires (Figure 4). Finally, both the elbow and wrist were immobilized with a long-arm plaster splint.

Postoperative evaluation

**Figure 5:**
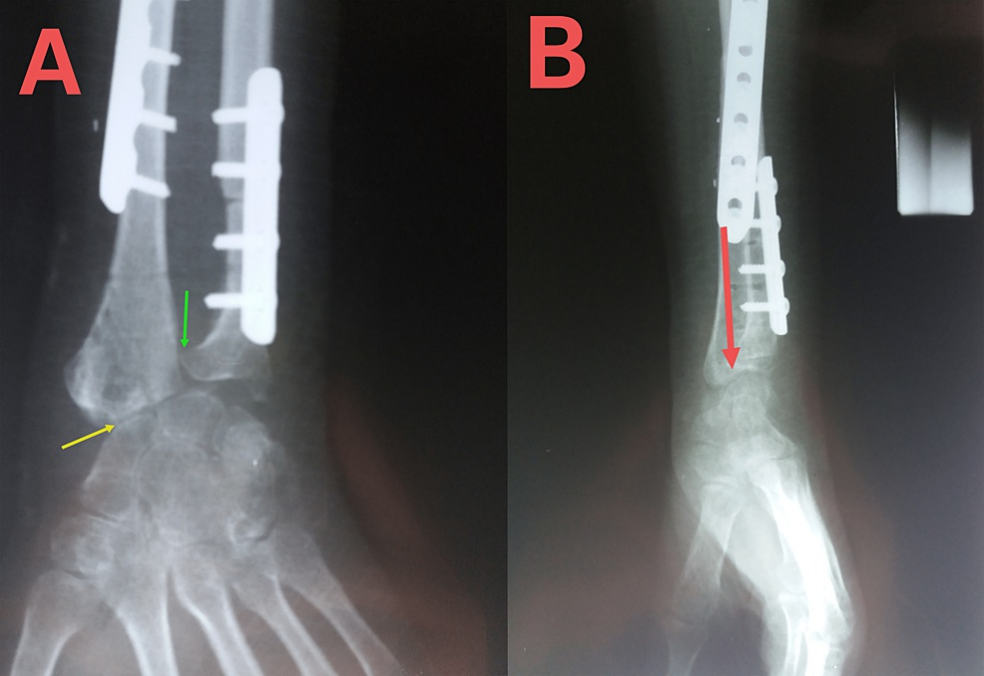
X-rays three months after the surgery. (A) Anteroposterior view, normal radio-ulnar variance (green arrow), good articulation between the graft and the proximal row of the wrist bone (yellow arrow); (B) Lateral view, good lateral alignment of the graft to wrist bones (red arrow).

The postoperative assessment involved monitoring bone healing through a comparison of preoperative and follow-up radiographs. The splint was removed six weeks post surgery, and K-wires were extracted after 12 weeks, as illustrated in Figure 5. At this juncture, the patient received clearance for gentle range-of-motion exercises. Wrist function was evaluated during follow-up clinical examinations. Internal fixation removal occurred 18 months post-surgery. Additionally, complications in the donor's knee were assessed.

**Figure 6:**
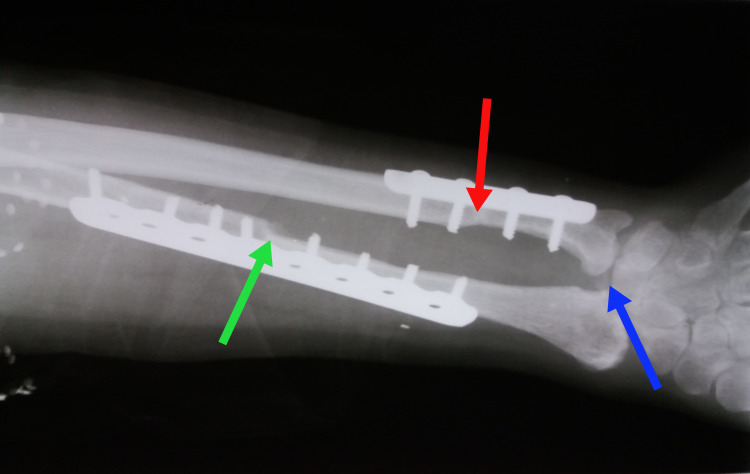
X-ray at the one-year follow-up showing good union of the graft with residual radius (green arrow), complete union of shortened ulna (red arrow), and the moderate distraction of radioulnar joint (blue arrow).

The patient remained under observation for three years, and a bony union of the graft was observed after 12 months (Figure 6). The stability of the wrist was deemed acceptable, and the range of motion was as follows: wrist dorsal extension 0̊-30̊, wrist palmar flexion 0̊-10̊, radial deviation 0̊-10̊, ulnar deviation 0̊-15̊, pronation 0̊-25̊, and supination 0̊-20̊. 

## Discussion

The successful treatment of GCTs of the distal radius is a challenging task due to the complex structures surrounding the affected area. In advanced stages of the disease, where the tumor has invaded the wrist joint, en bloc resection is often recommended to minimize the chances of recurrence. However, such a procedure often results in large defects that pose significant challenges to the restoration of wrist function [8,9].

The restoration of wrist function is hindered by large defects. However, the proximal fibula on the same side of the body can serve as a suitable replacement for the distal radius due to its similar shape and curvature to the proximal carpal row. The recurrence rates for this procedure have been reported to be between 0% and 25% [10,11]. Out of 16 patients in one series, 10 developed wrist subluxation [12], while in another series of 15 patients treated with plate fixation, two developed severe wrist subluxation causing significant pain, deformity, and loss of function [13]. Nevertheless, in a study using a non-vascularised free fibular graft for recurrent GCT of the distal radius, no wrist subluxation was reported [14]. However, in a study by Murray and Schlafly, five among 18 patients developed nonunion due to inadequate fixation of the fibular graft [11].

In a separate study involving 10 patients using free non-vascularised fibular graft for post-traumatic lower limb bone loss, the union rate was 100% [15]. According to a study involving 12 patients who received wide resection of the distal radius and reconstruction using non-vascularised autogenous fibular grafts for bone GCT, it was found that this method achieved favorable cosmetic and functional results in restoring the distal radius [16].

The Syrian conflict has presented many challenges for medical professionals, including limited access to medical resources and equipment. Despite these challenges, we were able to perform a rare and difficult surgery on a patient with a GCT in their distal radius. GCTs are benign tumors that can be invasive and can sometimes metastasize to the lungs. These tumors are typically found in the epiphyses of tubular bones, but they can also affect flat bones and the spine.

In our case, the GCT was located in the distal radius and had degenerated into an aneurysmal bone cyst, resulting in a large bulge in the distal radius and medial dislocation of the distal ulna. Surgical options for this type of tumor are limited due to its size and the thinning of the cortex. En-bloc resection is a good option, but the challenge, in this case, was how to resect the tumor and reconstruct the bone defect and radio-ulnar variance while restoring movement in the wrist joint in a conflict region where no oncology center was available.

Despite the limited resources, we decided to use an ipsilateral non-vascularized proximal fibula as a graft to reconstruct the bone defect. The fibular head morphologically resembles the distal radius, and its articular surface can articulate with the proximal row of wrist bones to reconstruct the wrist joint. The procedure involved the harvesting of the graft from the fibula after inflating a tourniquet and making a lateral incision over the fibular head. We then disarticulated the proximal tibiofibular joint and cut the insertion of the biceps femoris muscle and lateral collateral ligament from the fibular head to osteotomize the fibula 10 cm distal to the radial head. The graft was then prepared and stabilized using DCP and K-Wires, and the patient was placed under observation for one year.

After 12 months of follow-up, X-rays showed good union of the fibular graft and residual radius, good union in the site of shortening ulna, and mild radio-ulnar joint distraction. The radio-ulnar variance and wrist range of motion were acceptable. Our success in this surgery is particularly noteworthy given that it was carried out during the Syrian conflict when the healthcare system was severely compromised. Despite the challenging circumstances, we were able to provide our patient with the best possible care and achieve a positive outcome. We believe that our experience demonstrates the importance of resourcefulness, adaptability, and resilience in the face of adversity.

## Conclusions

This report outlined a comprehensive diagnostic approach utilizing plain radiographs, CT scans, and histopathological examination to confirm a grade III GCT of the distal radius. Despite the formidable challenges presented by the Syrian conflict, our surgical intervention involving en bloc resection and reconstruction with an ipsilateral proximal fibular graft proved effective in restoring wrist function. This underscores the importance of adaptability and resourcefulness in delivering optimal medical care in conflict zones. Our approach not only addresses the diagnostic and therapeutic aspects of GCT but also highlights the resilience and ingenuity required to navigate complex healthcare scenarios in war-torn regions.

## References

[1] Dahlin DC, Cupps RE, Johnson EW Jr (1970). Giant-cell tumor: a study of 195 cases. Cancer.

[2] Viswanathan S, Jambhekar NA (2010). Metastatic giant cell tumor of bone: are there associated factors and best treatment modalities?. Clin Orthop Relat Res.

[3] Zhang Q, Zhao H, Maheshwari AV, Cai L, Yu F, Niu X (2010). Isolated cardiac metastasis from a histologically "benign" giant-cell tumor of the distal end of the femur: a case report. J Bone Joint Surg Am.

[4] Vander Griend RA, Funderburk CH (1993). The treatment of giant-cell tumors of the distal part of the radius. J Bone Joint Surg Am.

[5] Pazionis TJ, Alradwan H, Deheshi BM, Turcotte R, Farrokhyar F, Ghert M (2013). A systematic review and meta-analysis of en-bloc vs intralesional resection for giant cell tumor of bone of the distal radius. Open Orthop J.

[6] He H, Zeng H, Luo W, Liu Y, Zhang C, Liu Q (2019). Surgical treatment options for giant cell tumors of bone around the knee joint: extended curettage or segmental resection?. Front Oncol.

[7] Maniar MH, Mankar S, Sakhre R (2023). En bloc resection with reconstruction using a customized megaprosthesis in a case of proximal humerus giant cell tumor: a case report. Cureus.

[8] Harness NG, Mankin HJ (2004). Giant-cell tumor of the distal forearm. J Hand Surg Am.

[9] Maruthainar N, Zambakidis C, Harper G, Calder D, Cannon SR, Briggs TW (2002). Functional outcome following excision of tumours of the distal radius and reconstruction by autologous non-vascularized osteoarticular fibula grafting. J Hand Surg Br.

[10] Cheng CY, Shih HN, Hsu KY, Hsu RW (2001). Treatment of giant cell tumor of the distal radius. Clin Orthop Relat Res.

[11] Murray JA, Schlafly B (1986). Giant-cell tumors in the distal end of the radius. Treatment by resection and fibular autograft interpositional arthrodesis. J Bone Joint Surg Am.

[12] Dhamni IK, Jain AK, Maheswari V, Singh MP (2005). Giant cell tumours of the lower end of radius: problems and solutions. Indian J Orthop.

[13] Saraf SK, Goel SC (2005). Complications of resection and reconstruction in giant cell tumour of distal end of radius- an analysis. Indian J Orthop.

[14] Lawal YZ, Garba ES, Ogirima MO, Dahiru IL, Maitama MI, Abubakar K, Ejagwulu FS (2011). Use of non-vascularized autologous fibula strut graft in the treatment of segmental bone loss. Ann Afr Med.

[15] Lin KC, Tarng YW, Hsu CJ, Renn JH (2014). Free non-vascularized fibular strut bone graft for treatment of post-traumatic lower extremity large bone loss. Eur J Orthop Surg Traumatol.

[16] Humail SM, Ghulam MK, Zaidi IH (2014). Reconstruction of the distal radius with non-vascularised fibular graft after resection of giant cell tumour of bone. J Orthop Surg (Hong Kong).

